# Electrospun Nanofibers including Organic/Inorganic Nanohybrids: Polystyrene- and Clay-Based Architectures in Immunosensor Preparation for Serum Amyloid A

**DOI:** 10.3390/bios13070673

**Published:** 2023-06-23

**Authors:** Gizem Evren, Eray Er, Esra Evrim Yalcinkaya, Nesrin Horzum, Dilek Odaci

**Affiliations:** 1 Department of Biochemistry, Faculty of Science, Ege University, Bornova, Izmir 35100, Turkey; gizemmevrennn@gmail.com (G.E.); eray_1063@hotmail.com (E.E.); 2 Department of Chemistry, Faculty of Science, Ege University, Bornova, Izmir 35100, Turkey; esra.evrim.saka@ege.edu.tr; 3 Department of Engineering Sciences, Izmir Katip Celebi University, Cigli, Izmir 35620, Turkey; nesrin.horzum.polat@ikc.edu.tr

**Keywords:** nanobiotechnology, serum amyloid A, nanofiber, clay, dendrimer, immunosensor

## Abstract

Diagnostic techniques based on biomolecules have application potential that can be realized in many fields, such as disease diagnosis, bioprocess imaging, food/beverage industries, and environmental pollutant imaging. Successful surface immobilization of biomolecules is critical to increasing the stabilization, sensitivity, and selectivity of biomolecules used in bioassay systems. Nanofibers are good candidates for the immobilization of biomolecules owing to many advantages such as morphology and pore size. In this study, montmorillonite (MMT) clay is modified with poly(amidoamine) (PAMAM) generation 3 (PAMAM_G3_) and added to polystyrene (PS) solutions, following which PS/MMT-PAMAM_G3_ nanofibers are obtained using the electrospinning method. The nanofibers are obtained by testing PS% (wt%) and MMT-PAMAM_G3_% (wt%) ratios and characterized with scanning electron microscopy. Antiserum amyloid A antibody (Anti-SAA) is then conjugated to the nanofibers on the electrode surface via covalent bonds using a zero-length cross linker. Finally, the obtained selective surface is used for electrochemical determination of serum amyloid A (SAA) levels. The linear range of PS/MMT-PAMAM/Anti-SAA is between 1 and 200 ng/mL SAA, and the detection limit is 0.57 ng/mL SAA. The applicability of PS/MMT-PAMAM_G3_/Anti-SAA is investigated by taking measurements in synthetic saliva and serum both containing SAA.

## 1. Introduction

The acute-phase response (APR) is a nonspecific innate reaction of the body to local or systemic disturbances in homeostasis that occurs due to infections, inflammations, stresses, or immunological disorders [1]. During APR, besides fever and hormonal changes, serum protein levels might change. Among serum proteins, serum amyloid A (SAA) and C-reactive protein (CRP), mainly secreted from the liver, are important biomarkers in predicting certain diseases [2]. SAA, a family of apolipoproteins defined as acute-phase proteins, has a precursor relationship with the main components of amyloid A fibril in reactive amyloidosis. It performs various roles, including the transport of cholesterol to the liver for secretion into the bile, recruitment of immune cells to inflammatory areas, chemotaxis of leukocytes, immunomodulatory activity, opsonization, and induction of enzymes that degrade the extracellular matrix [3,4]. SAA has also been reported to be a better biomarker than CRP for diseases except for inflammatory bowel disease [5]. Many comparative studies have been reported, such as those on monitoring the course of coronavirus disease 2019 [6,7] and inflammatory rheumatic diseases [8] and predicting mucosal healing in patients with ulcerative colitis in clinical remission [9]. Therefore, we must develop a rapid and sensitive method for the detection of SAA in human serum. A variety of means based on the surface plasmon resonance method [10], enzyme-linked immunosorbent assay (ELISA) [11], mass spectrometry [12], latex particle-enhanced immunoturbidimetry [13], immunonephelometry [14], and electrochemical immunosensors [15] has been applied for SAA detection. However, ELISA is complicated, expensive, and labor-intensive, and therefore, common detection methods based on ELISA are time-consuming. Electrochemical immunosensors are potential alternatives for highly sensitive, cost-effective, and straightforward detection of target molecules while overcoming the abovementioned limitations [16,17,18,19,20]. For example, Xia et al. developed a highly sensitive electrochemical immunological biosensor for SAA by modifying glassy carbon electrodes (GCEs) using carboxy-end-capped polypyrrole (PPy-α-COOH), multiwalled carbon nanotubes (MWCNTS), ionic liquid, and chitosan (Chit). On the one hand, PPy-α-COOH provides binding sites for covalent immobilization of Anti-SAA; on the other hand, MWCNTS/IL/Chit nanocomposites provide satisfactory electrical conductivity as well as high stability [15]. Ultralow amounts of analytes might be detected with electrochemical biosensors by means of signal amplification based on bioelectrocatalytic reactions, nanomaterial labeling, molecular biological strategies, etc. [21,22]. Moreover, the surface of the electrodes is a key point affecting the response of electrochemical immunosensors [23]. Conducting polymers [24], natural polymers [25], inorganic materials [26], and nanostructures [27] are used for surface modification to prepare immunosensors. Among them, electrospun nanofibers have attracted considerable attention because of their large surface area, controllable film thickness, and ease of forming functional groups on their surface.

The large surface area of electrospun nanofibers are combined with clay, an ideal inorganic structure that can be used as an immobilization matrix for biomolecules owing to its high absorption and cation-exchange capacity (CEC) and well-dispersive nature. Polymer/inorganic hybrid electrospun nanofibers can be fabricated using different types of modified clays and polymers [28,29,30]. Montmorillonite (MMT), the most preferred clay in analytical applications, has been modified using dendrimers [28,29,30], folic acid [31], hexadecyl trimethyl ammonium chloride [32], and sodium dodecyl sulfonate [33], and transformed into nanofibers with poly(vinyl) alcohol (PVA), poly(caprolacton) (PCL), and Chit, among others [28,29,30]. In our previous study, electrospun PVA nanofibers, including poly(amidoamine) generation 2 (PAMAM_G2_)-modified MMTs, were fabricated for the covalent immobilization of pyranose oxidase to prepare stable enzymatic glucose biosensors [29]. In other studies, electrospun polycaprolacton-Chit nanofibers, including PAMAM generation 0 (PAMAM_G0_) and PAMAM generation 1 (PAMAM_G1_)-modified MMTs, were coated with arginylglycylaspartic acid peptide and glutamate oxidase to prepare cell adhesion platforms and monosodium glutamate biosensors, respectively [28,30]. Herein, the surface of polystyrene (PS) nanofibers, as well as PAMAM_G3_-modified MMT, is considered to be an alternative to the routinely used ELISA platforms for the detection of SAA. Not only is the cost reduced but also the detection limit is improved. The sensor system is promising for the analysis of clinical serum samples owing to the ease of nanofiber fabrication and the advantages of electrochemical systems.

## 2. Materials and Methods

### 2.1. Materials

Polystyrene (PS), PAMAM dendrimer, ethylenediamine core, generation 3.0 solution ([NH_2_(CH_2_)_2_NH_2_]:(G = 3); dendriPAMAM(NH_2_)32) (ethylenediamine core, 20 wt.% in methanol; formula weight 6908.84 g/mol), potassium hexacyanoferrate (III) (K_3_[Fe(CN)_6_]), *N*-(3-dimethylaminopropyl)-*N′*-ethylcarbodiimide hydrochloride (EDC), and N-hydroxysuccinimide (NHS; 98%) were purchased from Sigma-Aldrich (St. Louis, MO, USA). *N*, *N*-Dimethylformamide (DMF; 99.8%), and serum amyloid A (SAA) were obtained from (Merck, Germany). Antiserum amyloid A (Anti-SAA) from OriGene (Rockville, MD, USA) and MMT from Southern Clay Products (Austin, TX, USA) were used. Synthetic serum and synthetic saliva were prepared for sample application experiments according to the literature. Briefly, the synthetic serum included KCl (4.5 mM), MgCl_2_ (1.6 mM), CaCl_2_ (5.0 mM), NaCl (145.0 mM), urea (2.5 mM), D(+)-glucose (4.7 mM), and albumin (0.1 mM) [34]. The synthetic saliva included KCl (13.0 mM), KSCN (20.0 mM), CaCl_2_.H_2_O (1.5 mM), NaCl (2.2 mM), NH_4_Cl (1.82 mM), KH_2_PO_4_ (48.0 mM), NaHCO_3_ (67.5 mM), Na_2_SO_4_.10H_2_O (2.4 mM), and urea (33.0 mM) [35].

### 2.2. Apparatus

Electrospun nanofibers were obtained on indium tin oxide glasses (Teknoma, IzmirTurkey) using the electrospinning unit NanoWeb Electrospin 103 (MaviTech, Mersin-Turkey). Notably, the samples had been sonicated for 1 h in an ultrasonic mixer device (Medisson Ultrasonic Clenar, Istanbul-Turkey) before obtaining the nanofibers in the electrospinning device. The contact angles of the nanofibers were measured using an Attention Theta goniometer. Cyclic voltammetry (CV), differential pulse voltammetry (DPV), and electrochemical impedance spectroscopy (EIS) measurements were performed using a PalmSens4 potentiostat (PalmSens Instruments, Houten, The Netherlands) at room temperature. The structures of neat MMT and MMT-PAMAM_G3_ were analyzed with Fourier-transform infrared spectroscopy using the Perkin Elmer Pyris 1 spectrometer (USA) in the 400–4000 cm^−1^ range. Pellets were prepared by dispersing the samples into KBr for the Fouirer Transform Infrared Spektrofotometric (FTIR) analysis of the clay samples. Additionally, an attenuated total reflectance apparatus was used for the FTIR analysis of the nanofiber samples. The thermal behavior of the clay and organoclay samples was investigated using thermogravimetric analysis/differential thermogravimetry (TGA/DTG). The mass losses of the samples were detected at a heating rate of 10 °C/min and nitrogen gas flow rate of 75 mL/min in the temperature range of 25–800 °C. X-ray diffraction (XRD) patterns of the clay and organoclay samples were recorded on an XRD spectrometer (Philips E’xpert Pro, UK) equipped with a graphite monochromator using a Cu Kα radiation source (λ = 0.154 nm). The samples were scanned with a diffraction angle 2θ in the range of 4°–80° in 1° increments. The interlayer distances of the homogenized powder clay and organoclay samples were determined from the diffraction peaks using the Bragg equation (nλ = 2dsinθ). The zeta potential of the MMT particles was measured using a Zeta-Meter 3.0+ with Zeiss DR microscope, GT-2 type quartz cell, molybdenum cylinder anode, and platinum rod cathode electrode. Energy-dispersive X-ray (EDX) spectra and SEM micrographs were obtained on a Carl Zeiss 300 VP SEM at an accelerating voltage of 7 kV. X-ray photoelectron spectroscopy (XPS) analysis was performed on each of the nanofibers using Thermo Scientific K-Alpha.

### 2.3. Preparation of MMT-PAMAM_G3_

MMT with an organic molecule, PAMAM_G3_, was modified according to a cation-exchange process [28,29,30]. This process is based on the replacement of hydrated Na^+^ ions in the interlayers of clay minerals with PAMAM_G3_ containing quaternary alkyl ammonium ions. According to this process, 1.0 g of MMT was dispersed in deionized water at room temperature using a mechanical stirrer. Meanwhile, PAMAM_G3_ equivalent to approximately twice the CEC of MMT was dissolved in deionized water. Subsequently, PAMAM_G3_ was treated with 1.0 M aqueous HCL solution to obtain an alkyl ammonium salt. This PAMAM_G3_ solution was gradually added to the dispersion containing clay particles, and a displacement reaction was performed at room temperature for 24 h using a magnetic stirrer. The obtained organoclay (MMT-PAMAM_G3_) was precipitated using centrifugation (18,000 rpm, 15 min) and repeatedly washed with deionized water and ether until no halide ions were detected upon adding 0.1 M aqueous AgNO_3_ solution. The final product was dried in a vacuum oven for 2 days at 45 °C.

### 2.4. Fabrication of PS/MMT-PAMAM_G3_ Electrospun Nanofibers

Electrospinning solutions were prepared by dissolving 20 wt% PS beads in DMF at 50 °C. Subsequently, MMT-PAMAM_G3_ (0.5 wt%) and Triton X100 (2.5 wt%) were added to this solution. After stirring overnight and applying ultrasonication for 4 h, the polymer solution was filled into a 2 mL syringe with 0.8 mm diameter and placed in a microinfusion pump. The working conditions were optimized as follows: an applied voltage of 8–15 kV, a distance between the needle and metal collector of 10–15 cm, and a flow rate of 0.8–2 mL/h. The PS/MMT-PAMAM_G3_ nanofibers were collected on the GCEs after 45–60 s. The electrodes were left overnight at room temperature to dry their surfaces. During the fabrication of the nanofibers, the temperature was 20–25 °C and the humidity between 60% and 65%.

### 2.5. Preparation of PS/MMT-PAMAM_G3_/Anti-SAA Immonusensor

Total amounts of 0.2 M EDC and 0.4 M NHS were dissolved in phosphate-buffered saline (PBS) to conjugate Anti-SAA on an electrode surface covered with the PS/MMT-PAMAM_G3_ nanofibers. Anti-SAA:EDC:NHS (1:1:2; *v*:*v*:*v*) was mixed in PBS and incubated at 1200 rpm for 15 min. The mixture was added onto the PS/MMT-PAMAM_G3_ nanofiber-covered electrode surface and incubated for 4h. Following this, the PS/MMT-PAMAM_G3_/Anti-SAA was rinsed with buffer. EIS, CV, and DPV were performed to confirm that the GCE surface had been modified with PS/MMT-PAMAM_G3_ and PS/MMT-PAMAM_G3_/Anti-SAA. A 0.164 g mass of hexacyanoferrate (III) (K_3_[Fe(CN)_6_]) (HCF) was dissolved in 1.0 mL of PBS, and KCl (3.73 g) was dissolved in 50 mL of distilled water. HCF (100 μL, 5 mM) and KCl (1 mL, 0.1 M) were added to 8.9 mL of PBS buffer in the chamber. Mixing was achieved with the help of a magnetic bar. Protein determination was performed with Bradford to calculate the amount of antibody bound to the surface of the PS/MMT-PAMAM_G3_ nanofibers [36]. First, the protein standard graph was plotted. Then the amount of free antibody and unbound antibody was calculated; hence, the amount of antibody on the surface of the PS/MMT-PAMAM_G3_/Anti-SAA nanofibers was calculated. Then, the molecular weight of the antibody was kept constant as 12 kDa and the number of antibodies on the surface was calculated according to the literature [37,38].

### 2.6. Electrochemical Measurements

PalmSens4 was used as a potentiostat. A triple-electrode system consisting of Ag/AgCl as the reference electrode, Pt counter electrode as the auxiliary electrode, and GCE as the working electrode was used. The measurement was performed in a nonmixing environment. CV and DPV measurements were taken in a potential range of −0.4 Volts (V) and +0.8 Volts (V). EIS measurements were taken with 0.18 V as the excitation voltage, 0.21 × 10^−4^–100 kHz frequency, and 10 mV dc potential.

## 3. Results

### 3.1. Characterization of MMT-PAMAM_G3_

**Figure 1:**
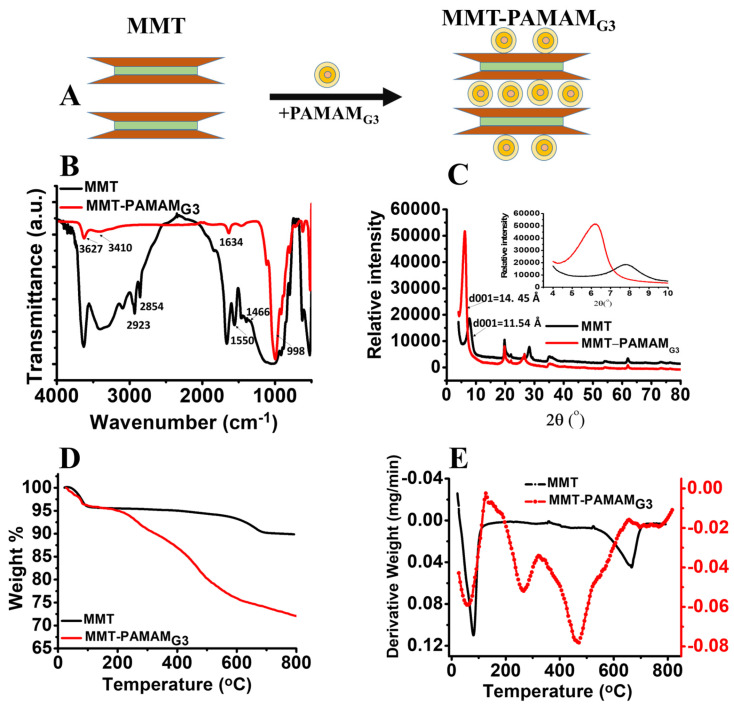
(**A**) Intercalation of poly(amidoamine) generation 3 (PAMAM_G3_) into the montmorillonite (MMT) sheets. (**B**) Fouirer transform infrared (FTIR) spectra, (**C**) X-Ray diffraction (XRD) pattern (inset shows XRD pattern with 2θ = 3–10 scale), (**D**) Thermogravimetric (TG) thermogram, (**E**) Differential thermogravimetric (DTG) thermogram of MMT and MMT-PAMAM_G3_.

PAMAM is a class of hyperbranched polymers with functional groups and appropriate for the covalent conjugation of biological molecules [39]. MMT was modified with PAMAM_G3_ dendrimers to obtain organoclay. For this purpose, PAMAM_G3_ was treated in an acidic medium to form an alkyl ammonium salt and intercalated into the layers of the clay minerals. Figure 1A shows the preparation of MMT-PAMAM_G3_. The MMT and MMT-PAMAM_G3_ samples were structurally characterized with FTIR and XRD analyses. Additionally, the thermal stability of the samples was determined using TGA/DTG. The eta potentials were measured for the charge changes of pure clay and organoclay samples. The FTIR spectra of neat MMT and organomodified MMT are shown in Figure 1B. In the FTIR spectrum of pure MMT pellets, the characteristic O–H stretching vibration of H_2_O owing to hydration in the structure shows a strong band at 3410 cm^−1^. Additionally, the O-H vibration band due to the relative humidity in the building is seen at 3627 cm^−1^. Another strong band is observed at approximately 1634 cm^−1^ due to O-H bending vibrations of hydrated water molecules. A characteristic strong band at approximately 998 cm^−1^ was attributed to the Si–O–Si and Si–O–Al stretching. Besides the peaks of pure MMT, some new clear bands were observed in the FTIR spectrum after modification of MMT with PAMAM_G3_, owing to the existence of dendrimers. Significant bands of–CH stretching vibrations were observed at 2923 and 2854 cm^−1^, and those of–CH_2_ and–CH bending vibrations were observed at 1466 and 1400 cm^−1^, respectively [40]. The band of the PAMAM_G3_ N-H amine group was clearly designated at approximately 1550 cm^−1^. These new organic bands supported that alkyl ammonium ions were intercalated between the layers and also adsorbed on the surface. The XRD technique is frequently used to determine the distance between layers of clay minerals composed of a layered structure. Information regarding the crystal planes of the clay particles is given by measuring the gallery height and basal spacing (d001) of the stacked clay layers. Upon modification, organic molecules were replaced by Na^+^ ions between the layers. Consequently, the peak of the diffraction angle shifted toward lower angles with increasing interlayer distance. The XRD patterns for pure MMT and MMT-PAMAM_G3_ are shown in Figure 1C. For pure MMT, characteristic diffraction peaks are seen at 2θ = 7.73°, which corresponds to the basal interval as d001 = 11.42 Å using the Bragg equation. The d-spacing of the (d001) basal plane represents the sum of the thickness of MMT layers and the heights of the galleries. This value depends on the size of the cations and the presence of interlaminar water. The replaceable cations in the clay mineral were replaced with alkylammonium cations (PAMAM_G3_ ammonium cations) to prepare the organoclays, increasing the d001 value. From the XRD pattern of MMT-PAMAM_G3_, a maximum diffraction peak can be seen at 2θ = 6.01°. The basal spacing value (d001) was calculated to be 14.71 Å from the Bragg equation. The increment in the d range (3.29 Å) indicates that the alkylammonium cations were intercalated between the silicate layers. This result is compatible with that of our previous study on PAMAM with variable molecular weights [28,29,30]. The basal spacing value of interlayers increases with the chain length and also the molecular weight of PAMAM.

The thermal behaviors of the samples were compared using TGA/DTG analysis. The TG/DTG curves for MMT and organomodified MMT (MMT-PAMAM_G3_) are shown in Figure 1D,E; the weight losses in the TGA curve correspond to the temperature range from room temperature to 800 °C. For pure MMT, a 9.9% weight loss is observed in this temperature range owing to the thermal degradation of the structure. Further examination is obtained from the DTG thermogram (see Figure 1E). The weight losses were observed in two main temperature zones: below 200 °C and above 600 °C. The first step, observed at 90 °C, is attributed to the dehydration of the water physically adsorbed, and the second step, observed at 667 °C, is attributed to the dehydroxylation of water molecules retained by the exchangeable cations in MMT. The weight loss of MMT-PAMAM_G3_ increased to 27.8% in the same temperature range after the exchange with alkylammonium ions. This increase in weight loss demonstrated that the PAMAM_G3_ organic molecules were both adsorbed on the clay surface and bonded between layers. The results from the detailed analysis of the DTG thermogram of MMT-PAMAM_G3_ showed that the degradation peak of PAMAM_G3_ as an organic molecule was observed at approximately 265 °C. The thermal decomposition of MMT-PAMAM_G3_ occurred in three steps. The first zone was located below 150 °C due to the removal of adsorbed water and gaseous species. The second zone was between 200 °C and 300 °C, and the third zone corresponded to the temperature range of 400–500 °C, which is attributed to the dehydroxylation of structural –OH units from organoclay [41]. This degradation peak was shifted to a lower temperature according to pure MMT (667 °C for MMT; 460 °C for MMT-PAMAM_G3_). This shifted degradation peak confirmed the bonding between the organic molecule (PAMAM_G3_) and clay mineral (MMT). The zeta potential is an impressive index of the magnitude of interaction between colloidal particles such as clay minerals. The magnitude of zeta potential indicates the potential stability of a colloidal system and is also a sign of the surface charge. MMT clay mineral has a negative charge and, thus, possesses a negative zeta potential. The zeta potentials of MMT and MMT-PAMAM_G3_ were measured to confirm the changes in surface properties. The zeta potential of pure MMT was measured to be −41 ± 2 mV [42]. After the modification step, the zeta potential of MMT-PAMAM_G3_ was a less-negative value of −25 ± 1 mV due to the absorption of the positively charged PAMAM_G3_ salt at the surface or in the interlayers of the mineral. The difference in zeta potential is compatible with our previous studies on different PAMAM dendrimers [28,29,30]. As expected, the zeta potential had fewer negative values with increasing surface functional groups and branching structure (−30 mV for the G0 dendrimer; −28 mV for the G_2_ dendrimer).

### 3.2. Characterization of the PS/MMT-PAMAM_G3_ Electrospun Nanofibers

**Figure 2:**
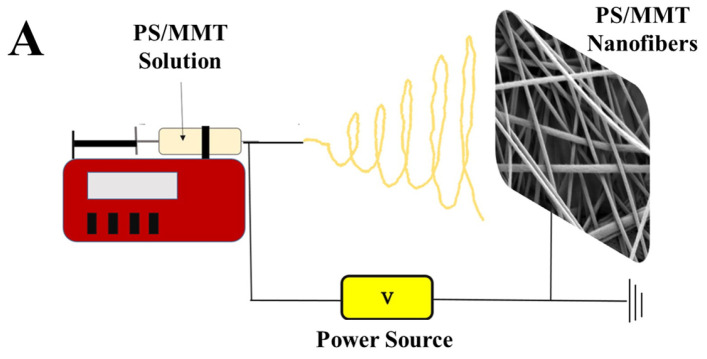

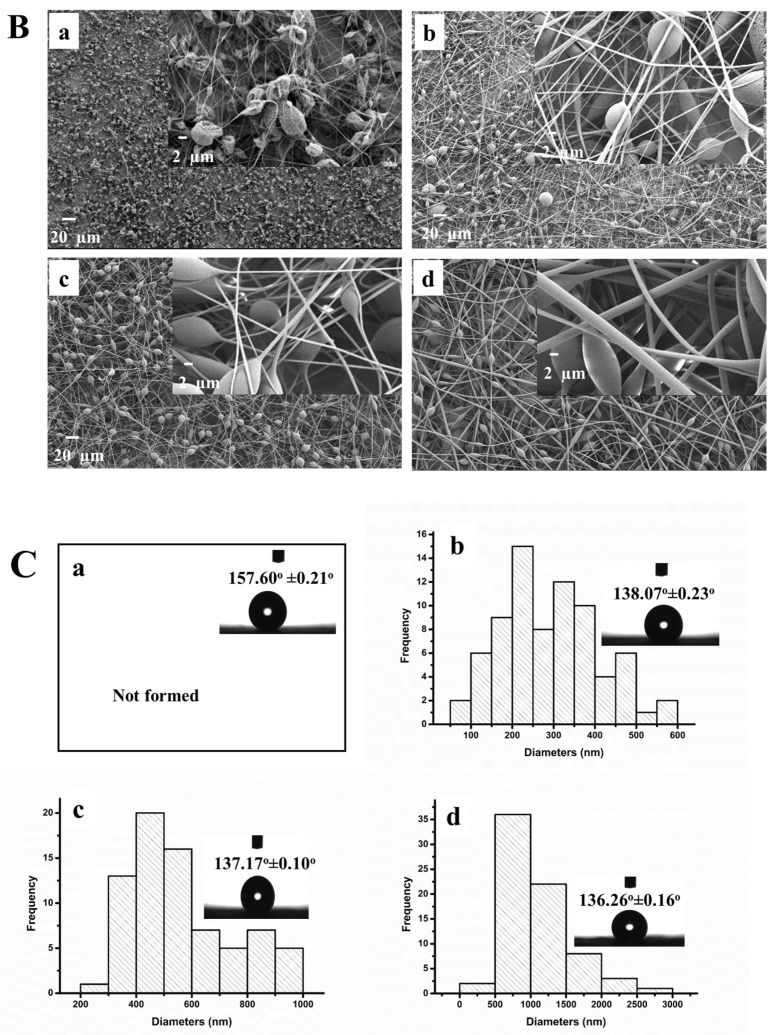
(**A**) Design of the polystyrene-based (PS) nanofiber matrix obtained by electrospinning. (**B**) Scanning electron microscopy (SEM) micrographs and (**C**) histograms of diameter distribution of the PS nanofibers fabricated from PS solutions with different concentrations (wt%): (**a**) 5%, (**b**) 10%, (**c**) 15%, and (**d**) 20% (insets of C show drop images of water and contact angles of the PS nanofibers).

The flow rate of the polymer solution, voltage, and distance between the syringe and collector must be optimized to obtain smooth nanofibers in the electrospinning technology. The impact on solvent-type PS nanofibers is considerable. Consequently, numerous solvents, including 1,2-dichloroethane, N,N-DMF, ethyl acetate, methyl ethyl ketone, tetrahydrofuran (THF), and even limonene, were studied for the electrospinning of PS [43,44,45]. Because of its higher boiling point, conductivity, and dielectric constant compared with other solvents, the widely used DMF was the most favored solvent for the synthesis of homogenous PS nanofibers with a smooth surface [46,47,48]. The concentration of the prepared solution is also one of the factors affecting the formation of smooth and bead-free nanofibers. Solutions containing PS at different concentrations were prepared and used to obtain PS nanofibers; this was to determine the appropriate polymer concentration in a bid to obtain bead-free nanofibers. A schematic representation of the design of PS-based nanofiber matrices is shown in Figure 2A. Morphological studies of the PS electrospun nanofibers, which were obtained using various PS concentrations, were conducted using an SEM. As shown in Figure 2B(a–d), the nanofiber morphology is not smooth and uniform for PS concentrations varying from 5% to 20%. Furthermore, nanofibers could not be obtained when a PS concentration of 25% and above was used. This is because the increase in PS concentration caused a higher viscosity and limited the flow of polymer solution from the syringe [49]. We thus decided to continue with 20% PS to form nanofibers because the nanofiber morphology tremendously changed as the PS concentration increased. The diameter distribution of the PS nanofibers is shown in Figure 2C(a–d). The diameter of the nanofibers prepared with 5% PS could not be measured because a considerable portion of them were beaded (Figure 2C(a)). The diameter of the nanofibers prepared in parallel with increasing PS concentration increased. The results obtained are consistent with those in the literature [50,51]. No major alteration was observed in the contact angles of the prepared nanofibers with increasing PS concentration, as shown in the insets of Figure 2C(a–d). We observed that the PS nanofibers prepared using different concentrations had highly hydrophobic properties. THF and DMF solvents were used in a study to obtain nanofibers (average diameter 400 nm) from 15% PS [52]. PS solutions of 5–35% were prepared with DMF solvent for electrospinning in another study [53]. Upon examining the results, while nanofibers could not be obtained from 5% PS, fibers could be synthesized in increasing PS concentrations. The diameter of the nanofibers also increased with increasing concentration.

**Figure 3:**
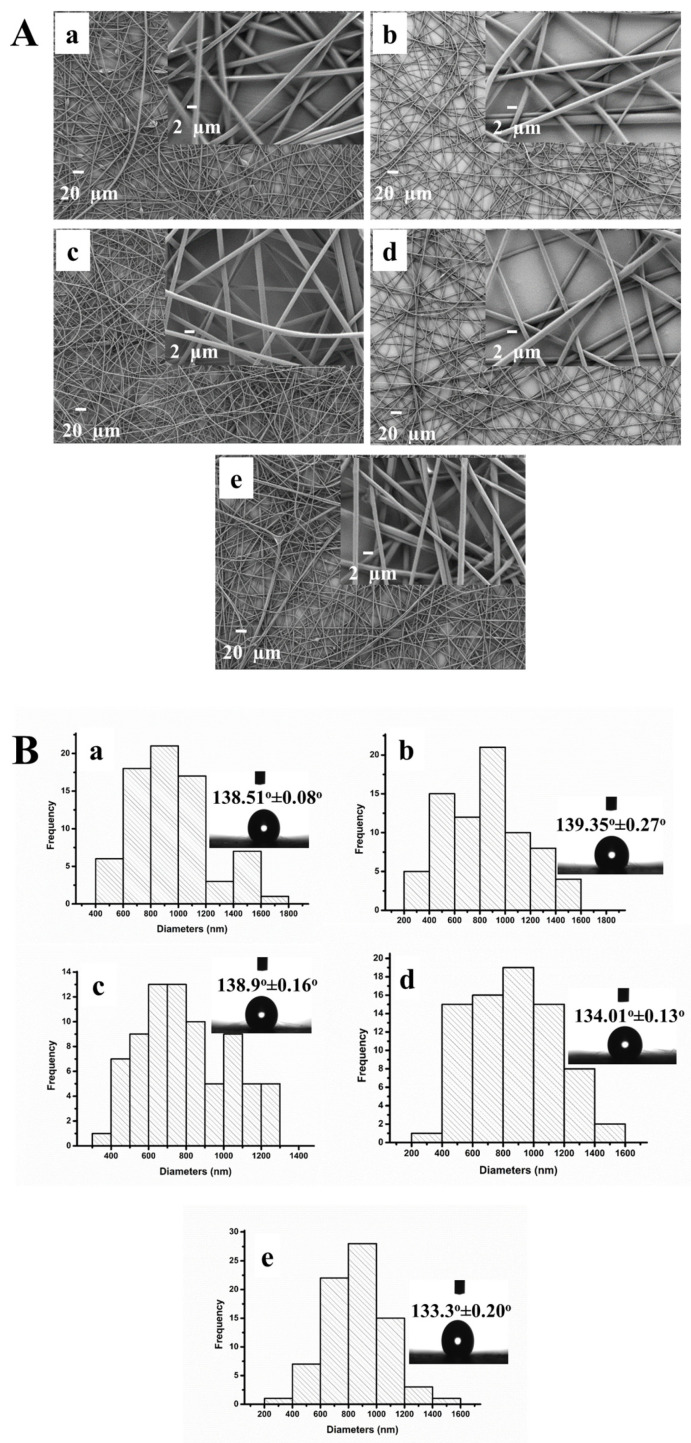
(**A**) SEM micrographs and (**B**) histograms of diameter distribution of the PS/MMT nanofibers containing different MMT concentrations (wt%) in 20% (wt%) PS: (**a**) 0.1%, (**b**) 0.25%, (**c**) 0.5%, (**d**) 1.0%, and (**e**) 2.0% (insets of B show drop images of water and contact angles of the PS/MMT nanofibers).

Upon deciding on a PS concentration of 20% (wt%), trials were performed to determine the concentration of clay to be used for preparing bead-free PS/MMT nanofibers. For this purpose, electrospun nanofibers were fabricated using a 20% (wt%) PS solution containing different MMT concentrations ranging from 0.1% to 2.0% (wt%). SEM micrographs of the PS/MMT nanofibers containing various amounts of MMT are shown in Figure 3A(a–e). The PS/MMT nanofibers prepared using PS solutions containing 0.25% (wt%) and 0.5% (wt%) MMT were considered suitable because they were more regular, thinner, and uniform. Histograms of the diameter distribution of the PS/MMT nanofibers are shown in Figure 3B(a–e). We observed no major differences in diameter among the PS/MMT nanofibers fabricated using different MMT concentrations. Adding MMT to the structure of the PS nanofibers did not change their contact angles and hydrophobicity, as shown in the insets of Figure 3B(a–e).

**Figure 4:**
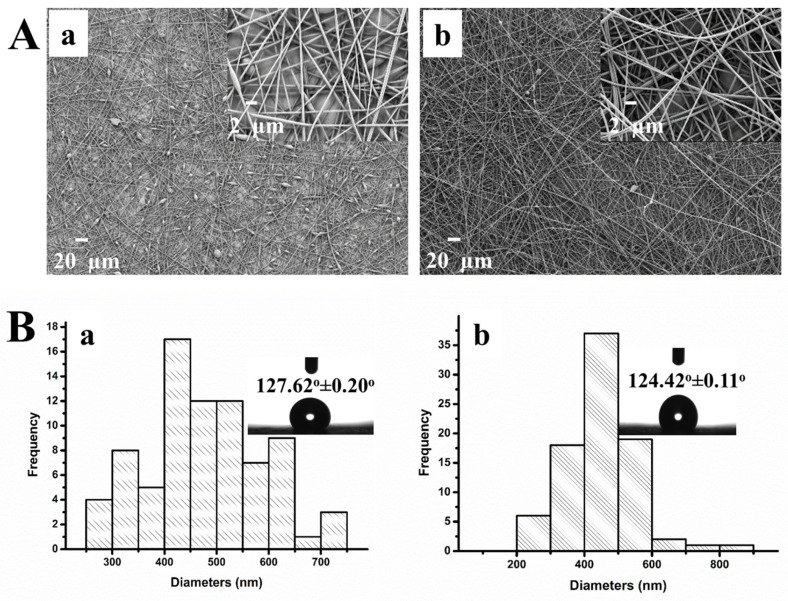
(**A**) SEM micrographs and (**B**) histograms of diameter distribution of the PS/MMT-PAMAM_G3_ nanofibers containing (**a**) 0.25% (wt%) and (**b**) 0.5% (wt%) MMT-PAMAM_G3_ in 20% (wt%) PS (insets of B show drop images of water and contact angles of the PS/MMT–PAMAM_G3_ nanofibers).

Because PS/MMT nanofibers containing 0.25% and 0.5% (wt%) MMT were smaller in diameter and easier to form, the same concentrations of MMT-PAMAM_G3_ were added to PS solutions. MMT-PAMAM_G3_ was synthesized and PS/MMT-PAMAM_G3_ nanofibers prepared to test the PAMAM_G3_ effect. Figure 4A(a,b) show the SEM micrographs of the PS/MMT-PAMAM_G3_ electrospun nanofibers prepared using PS solutions containing both concentrations of MMT-PAMAM_G3_ (i.e., 0.25% and 0.5% (wt%)). Histograms for diameter distributions of the PS/MMT-PAMAM_G3_ nanofibers are shown in Figure 4B(a,b). Evidently, PS/MMT-PAMAM_G3_ containing 0.5% (wt%) MMT-PAMAM_G3_ had the most homogeneous diameter distribution. However, the contact angles (insets of Figure 4B) and hydrophobicity of the PS/MMT-PAMAM_G3_ nanofibers did not change for either MMT-PAMAM_G3_ concentration. The optimum MMT-PAMAM_G3_ concentration in PS solution was selected to be 0.5% (wt%). This is because the resultant PS/MMT-PAMAM_G3_ nanofibers were bead-free, the diameter distribution of the nanofibers was better, and also the nanofiber diameters were smaller. The diameter of the PS, PS/MMT, and PS/MMT-PAMAM_G3_ nanofibers were 1115.54, 793.77, and 446.49 nm, respectively. The contact angles were (136.26 ± 0.16)°, (138.90 ± 0.16)°, and (124.42 ± 0.11)°, respectively. We can say that the addition of MMT and MMT-PAMAM_G3_ to the PS nanofiber structure decreased the diameter of the electrospun nanofibers. The addition of MMT did not affect the hydrophobicity of the PS nanofibers but the contact angles of PS/MMT-PAMAM_G3_ decreased after the addition of MMT-PAMAM_G3_ to the PS solution. The decrease can be explained by the NH_2_ dendrimer groups from the PAMAM_G3_ molecule intercalated into the MMT layers. In a study comparing the contact angles of PCL and PCL/PAMAM_G2_ nanofibers, the hydrophilic property was attributed to the NH_2_ terminal groups of the dendrimers and the OH groups generated upon aminolysis [54].

**Figure 5:**
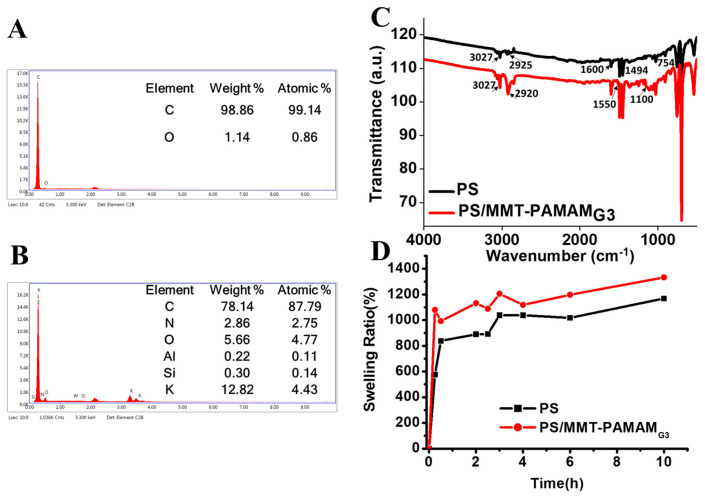
EDX results for (**A**) PS and (**B**) PS/MMT-PAMAM_G3_. (**C**) FTIR spectra of PS and PS/MMT-PAMAM_G3_. (**D**) Swelling ratios of the PS and PS/MMT-PAMAM_G3_ nanofibers.

Figure 5A,B show the EDX spectra of the PS and PS/MMT-PAMAM_G3_ nanofibers. The elemental compositions of the nanofibers confirmed the presence of dendrimer (N) and MMT (Al, Si, and K). We structurally characterized neat PS and PS/MMT-PAMAM_G3_ nanofibers with FTIR analysis (Figure 5C). In the FTIR spectrum of neat PS, absorption bands at 3027 and 2925 cm^−1^ corresponded to aromatic and aliphatic C-H stretching, respectively. The characteristic absorption bands of PS at 1600 and 1494 cm^−1^ can be attributed to C=C stretching of benzene rings. Additionally, the C-H deformation vibration band of benzene ring hydrogen appeared at 750 and 700 cm^−1^ [55]. The FTIR spectra of the PS/MMT-PAMAM_G3_ nanofibers were also investigated. In addition to characteristic PS bands, the increasing intensity of C-H stretching (3027 and 2920 cm^−1^) and a small new band at 1550 cm^−1^ (N-H stretching) can be attributed to the presence of PAMAM_G3_. Again, the new band at approximately 1100 cm^−1^ corresponds to the Si–O–Si and Si–O–Al stretching in MMT. Figure 5D shows the swelling characteristics (C) of the PS and PS/MMT-PAMAM_G3_ nanofibers. The swelling ratio results for 10 h showed a lower water uptake of the PS nanofibers compared with the PS/MMT-PAMAM_G3_ ones. The swelling ratio tended to increase with time and MMT-PAMAM_G3_ addition. PS nanofibers containing dendrimer-modified MMT had a high water uptake due to a higher amount of absorption groups on the surface. Simultaneously, with the addition of dendrimer-modified MMT, the decrease in the diameter of the nanofibers cause an increasing surface area of PS/MMT-PAMAM_G3_ (50 m^2^/g) compared to the PS nanofiber (45 m^2^/g). The presence of PAMAM also increases the water adsorption of PS/MMT-PAMAM_G3_ nanofibers. The water uptake of PS is connected with absorption in the amorphous regions and the porosity on/between the nanofibers, while the time-dependent increase can be explained by more time available for water penetration [54].

**Figure 6:**
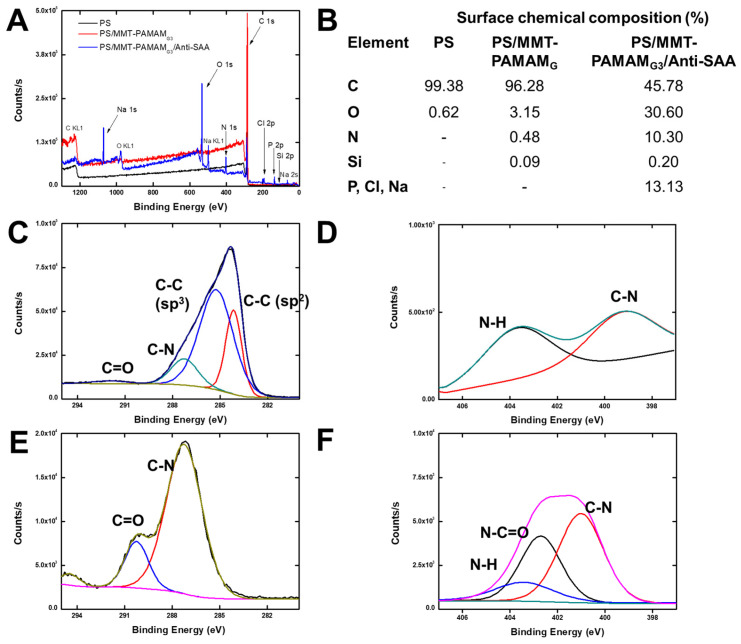
(**A**) XPS survey spectrum. (**B**) Percentage of surface chemical composition of PS, PS/MMT-PAMAMG_3_, and PS/MMT-PAMAMG_3_/Anti-SAA. (**C**) C1s and (**D**) N1s spectra of PS/MMT-PAMAMG_3_. (**E**) C1s and (**F**) N1s spectra of PS/MMT-PAMAMG_3_/Anti-SAA.

Figure 6A,B show the XPS survey spectra and surface chemical compositions, respectively, for PS, PS/MMT-PAMAM_G3_, and PS/MMT-PAMAM_G3_/Anti-SAA, indicating the presence of the antibody on the surface of the nanofibers. The C1s, N1s, and Si2p spectra of PS/MMT-PAMAM_G3_ are shown in Figure 6C–E, respectively. On the one hand, the characteristic peaks for PS/MMT-PAMAM_G3_ are observed from deconvoluted C1s spectra at 284.15 eV (C–C sp^2^), 285.24 eV (C–C sp^3^), 287.25 (C–N), and 291.93 eV (C–O) (Figure 6C). On the other hand, predominantly 287.25 (C–N) and 290.25 eV (C–O) peaks appeared for PS/MMT-PAMAM_G3_/Anti-SAA (Figure 6F). The fact that the carbon source is visible only from C–N and C–O indicates that the surface is completely covered with antibodies, because of which the PS/MMT-PAMAMG3 depth could not be reached and determined. The C–O peak shifted from 291.93 to 290.25 eV, C–N from 399.22 to 401.01 eV, and Si–O from 102.15 to 103.78 eV. The N1s and Si2p spectra show that PAMAM dendrons enable covalent linkage with biomolecules. PAMAM modified MMT, which provided free amine groups used for the covalent immobilization of Anti-SAA.

### 3.3. Characterization of the PS/MMT-PAMAM_G3_/Anti-SAA Immunosensor

**Figure 7:**
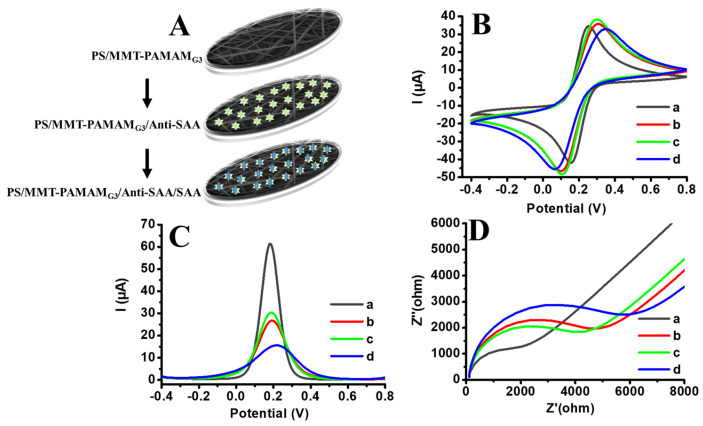
(**A**) Step-by-step modification of GCE surfaces. (**B**) CV, (**C**) DPV, and (**D**) Nyquist profile of (a) bare electrode, (b) PS/MMT-PAMAM_G3_, (c) PS/MMT-PAMAM_G3_/Anti-SAA, and (d) PS/MMT-PAMAM_G3_/Anti-SAA/SAA (Anti-SAA: 100 µg/mL and SAA: 2.0 ng/mL; scan rate: 50 mVs^−1^ (for CV and DPV) in 10 mL PBS (pH 7.4) containing 5.0 mM K_3_[Fe(CN)_6_] and 0.1 M KCl).

After having confirmed the success of the modification of MMT by PAMAM_G3_ and formation of nonbeaded, homogenous PS/MMT-PAMAM_G3_ electrospun nanofibers, Anti-SAA was immobilized on the PS/MMT-PAMAM_G3_ electrospun-nanofiber-modified GCE. Electrochemical characterization of PS/MMT-PAMAM_G3_/Anti-SAA immunosensor was performed using K_3_[Fe(CN)_6_] as a probe. Figure 7A shows the step-by-step modification of GCE surfaces. To characterize PS/MMT-PAMAM_G3_/Anti-SAA, the CV, DPV, and EIS techniques were applied to confirm the surface-modification steps of GCE by PS/MMT-PAMAM_G3_ and PS/MMT-PAMAM_G3_/Anti-SAA and then affinity-based binding of SAA on PS/MMT-PAMAM_G3_/Anti-SAA. According to the CV profiles (see Figure 7B), oxidation and reduction peaks of hexacyanoferrate(III) were detected and the I_anodic_ values were 34.58, 35.49, 38.23, and 32.01 µA for bare, PS/MMT-PAMAMG_3_-, PS/MMT-PAMAMG_3_/Anti-SAA-, and PS/MMT-PAMAM_G3_/Anti-SAA/SAA-modified GCE, respectively. The peak currents changed with the modification of the bare electrode with PS/MMT-PAMAM_G3_, PS/MMT-PAMAM_G3_/Anti-SAA, and PS/MMT-PAMAM_G3_/Anti-SAA/SAA. The calculated redox peak potential separations for bare, PS/MMT-PAMAM_G3_, PS/MMT-PAMAM_G3_/Anti-SAA-, and PS/MMT-PAMAM_G3_/Anti-SAA/SAA-modified GCE were 100, 212, 190, and 282 mV, respectively. The peak currents were obtained to be 62, 27, 31, and 16 µA, for the bare electrode, PS/MMT-PAMAM_G3_, PS/MMT-PAMAM_G3_/Anti-SAA, and PS/MMT-PAMAM_G3_/Anti-SAA/SAA, respectively, in the DPV trials (see Figure 7C). According to the DPV profile, the electrochemical oxidation peaks of hexacyanoferrate(III) decreased after the coating of GCE with PS/MMT-PAMAM_G3_ owing to the increased diffusion limitations of K_3_[Fe(CN)_6_] to the electrode surfaces. The current increased after the covalent immobilization of Anti-SAA on PS/MMT-PAMAM_G3_. Because antigen-binding fragments (Fab)_2_ are generally positively charged in the PBS medium at pH 7.4, electrostatic attraction occurred between K_3_[Fe(CN)_6_] and the PS/MMT-PAMAM_G3_/Anti-SAA-modified GCE, which caused an increased electron-transfer rate resulting in a higher electrochemical current [43]. The decrease in current was observed with the binding of SAA to the PS/MMT-PAMAM_G3_/Anti-SAA-modified GCE surface. This can be attributed to the increased diffusion limitations of K_3_[Fe(CN)_6_] to electrode surfaces. The EIS technique was applied, and the Nyquist semicircles obtained for the bare electrode, PS/MMT-PAMAM_G3_, PS/MMT-PAMAM_G3_/Anti-SAA, and PS/MMT-PAMAM_G3_/Anti-SAA/SAA are shown in Figure 7D. The charge transfer resistance (R_ct_) values for the bare electrode, PS/MMT-PAMAM_G3_-, PS/MMT-PAMAM_G3_/Anti-SAA-, and PS/MMT-PAMAM_G3_/Anti-SAA/SAA-modified GCE were 1677, 4025, 3541, and 5000 Ω, respectively. The insulator layer made on the GCE surface after successful modification steps resulted in changed R_ct_ values. These results are concomitant with the CV and DPV results.

### 3.4. Electrochemical SAA Detection Based on PS/MMT-PAMAM_G3_/Anti-SAA

**Figure 8:**
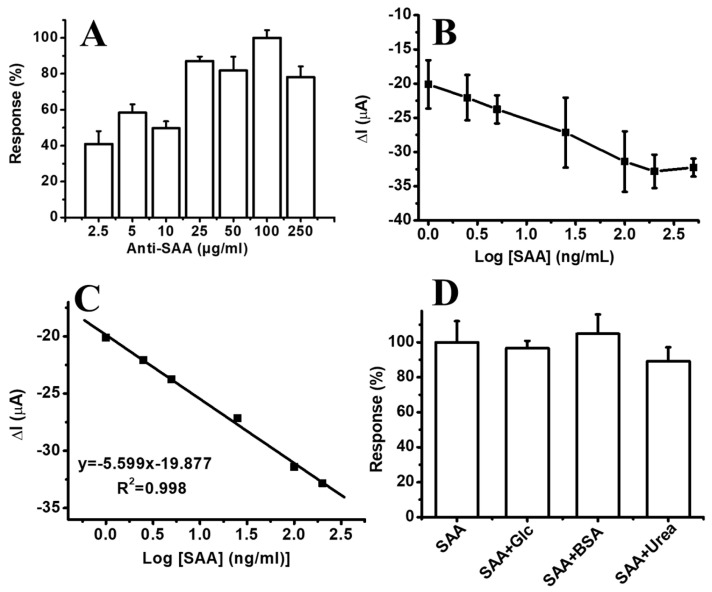
(**A**) Dependence of current on Anti-SAA concentration (in PBS pH 7.4; [SAA]: 50 ng/mL; n = 3). (**B**) Effect of SAA concentration on current response of PS/MMT-PAMAM_G3_/Anti-SAA (in PBS pH 7.4). (**C**) Linear range of PS/MMT-PAMAM_G3_/Anti-SAA for SAA detection (in PBS pH 7.4). (**D**) Effect of some potential interfering compounds on the current response of PS/MMT-PAMAM_G3_/Anti-SAA for SAA (in PBS pH 7.4; [SAA]: 25 ng/mL, [Glc]: 5.0 mM, [BSA]: 25 ng/mL, [urea]: 10 ng/mL).

Electrochemical experiments were performed in 50 mM PBS (pH 7.4) with 5.0 mM K_3_[Fe(CN)_6_] and 0.1 M KCl using the DPV technique. Trials were conducted to determine the optimum Anti-SAA concentration before SAA detection. For this purpose, the effect of various Anti-SAA concentrations on the sensor current was tested. Biofunctionalized surfaces were prepared using 2.5, 5.0, 10, 25, 50, 100, and 250 µg/mL Anti-SAA on PS/MMT-PAMAM_G3_. We observed from the results that the highest electrochemical current for the PS/MMT-PAMAM_G3_/Anti-SAA electrode was obtained using 100 µg/mL Anti-SAA (see Figure 8A). This was due to the full coverage of the PS/MMT-PAMAM_G3_ surface by Anti-SAA, leading to a higher response for Anti-SAA concentrations above 25 µg/mL. The antibody molecule number on the PS/MMT-PAMAM_G3_/Anti-SAA nanofiber-modified GCE which fabricated using 100 µg/mL Anti-SAA was calculated as 9.85x10^11^ Anti-SAA (diameter of GCE is 3.0 mm) and the immobilization yield was calculated as 73.91%. Because the highest current response of PS/MMT-PAMAM_G3_/Anti-SAA was observed using 100 µg/mL Anti-SAA, this concentration was used for further experiments as well. The effect of SAA on the current is shown in Figure 8B, and a linear range for SAA was obtained from 1.0 to 200 ng/mL with I(µA) = 5.299 [Log SAA] (ng/mL) + 19.877 (R^2^ = 0.998) (see Figure 8C). Based on the signal-to-noise ratios of eight trials, the limit of detection (LOD) was calculated by taking measurements with a 1.0 ng/mL SAA concentration, the lowest point of the calibration curve [56,57]. The LOD of PS/MMT-PAMAM_G3_/Anti-SAA was observed to be 0.57 ng/mL SAA. Notably, the SAA concentration should be below 3000 ng/mL in healthy adult individuals [58]. Five repeated measurements were taken with a 25 ng/mL SAA concentration, the midpoint of the calibration curve. The coefficient of variation was calculated to be 4.48%. Furthermore, the interference of other potential biological molecules was studied by testing the selectivity of the development of PS/MMT-PAMAM_G3_/Anti-SAA. For this purpose, we tested the effect of glucose, bovine serum albumin (BSA), and urea on the current response of PS/MMT-PAMAM_G3_/Anti-SAA. The designed PS/MMT-PAMAM_G3_/Anti-SAA exhibited a high response to SAA binding (see Figure 8D). Additionally, we observed no interference effect on the immunosensor response, on account of its high affinity to the Anti-SAA antibody. Table 1 presents the analytical characteristics of various sensors for SAA detection.

The samples were contaminated with 25 ng/mL SAA to feasibly determine PS/MMT-PAMAM_G3_/Anti-SAA for the detection of SAA in synthetic samples, including serum and saliva. The results obtained are presented in Table 2. 

## 4. Conclusions

A PS/MMT-PAMAM_G3_/Anti-SAA immunosensor was fabricated and tested for rapid and reliable detection of SAA without any interference in samples. First, MMT was modified with PAMAM_G3_, following which the synthesized MMT-PAMAM_G3_ was successfully combined with the PS polymer. Thereafter, the resulting PS/MMT-PAMAM_G3_ was tested as an alternative immobilization matrix for the binding of Anti-SAA in a bid to fabricate the SAA immunosensor. The implementation of the immunosensors was tested in different samples. The PS/MMT-PAMAM_G3_/Anti-SAA immunosensor afforded reliable results for the detection of SAA in physiological fluids.

## Figures and Tables

**Table 1:** The comparison of sensors for SAA detection.

Sensing Mode	Material	Recognition Molecule	Linear Range	LOD	Applied Sample	Ref
ELEC	MWCNTs MnO_2_NSs Co_3_O_4_NPs	MIP	0.12 pg/mL–12 μg/mL	0.12 pg/mL	Serum	[59]
LFA	Fe_3_O_4_@Au SERS tags	Anti-SAA	0.1–500 ng/mL	0.10 ng/mL	Blood	[60]
FL	Sandwich-type, antibody microarrays	Antibody	5.9–478 ng/mL	5.90 ng/mL	Serum	[61]
ELEC	MWCNTs/IL/Chit/GCE	Anti-SAA	0.001–900 ng/mL	0.30 pg/mL	Serum	[15]
FL	dQDs-FLISA	Anti-SAA	10–1000 ng/mL	2.39 ng/mL	Serum	[62]
ELEC	PS/MMT-PAMAM_G3_	Anti-SAA	1.0–200 ng/mL	0.57 ng/mL	Synthetic saliva and serum	This study

ELEC: electrochemistry; MWCNTs: multi-walled carbon nanotubes; MnO_2_NSs: manganese oxide nanospheres; Co_3_O_4_NPs: cobalt oxide nanoparticles; MIP: molecular imprinted polymer; LFA: lateral flow assay; Fe_3_O_4_: ferric oxide; Au: gold; SERS: surface-enhanced Raman scattering; FL: fluorescence; IL: ionic liquid; Chit: chitosan; dQDs-FLISA: dual quantum-dots-based fluorescence-linked immunosorbent assay.

**Table 2:** Application of PS/MMT-PAMAM_G3_ for the detection of SAA in artificial samples.

Synthetic Sample	Added SAA (ng/mL)	* Found SAA (ng/mL)	Recovery %
Saliva	25	24.82	99.29
Serum	25	24.35	97.41

* Trials were repeated three times.

## Data Availability

Not applicable.
